# The Design and Evaluation of Twisted HDPE Grass-Cutting Lines: A Performance Comparison with Commercial Nylon

**DOI:** 10.3390/polym17131804

**Published:** 2025-06-28

**Authors:** Anothai Pholsuwan, Wichain Chailad, Athapon Simpraditpan, Ekkachai Martwong, Kawita Chattrakul

**Affiliations:** 1 Department of Materials and Metallurgical Engineering, Faculty of Engineering, Rajamangala University of Technology Thanyaburi, Pathum Thani 12110, Thailand; anothai.p@en.rmutt.ac.th (A.P.); wichain_c@rmutt.ac.th (W.C.); athapon.s@en.rmutt.ac.th (A.S.); 2 Division of Science, Faculty of Science and Technology, Rajamangala University of Technology Suvarnabhumi, Phra Nakhon Si Ayutthaya 13000, Thailand

**Keywords:** grass-cutting machine, high-density polyethylene grass-cutting line, nylon grass-cutting line, cutting efficiency, tensile properties

## Abstract

This study presents the design and performance evaluation of a custom extrusion die for producing grass-cutting lines from high-density polyethylene (HDPE) with twist angles of 0°, 15°, 30°, and 45°. The mechanical properties, cutting efficiency, and energy consumption of the HDPE lines were compared with those of commercially available nylon lines with round and square profiles. The die successfully produced twisted HDPE lines with consistent geometry. Although the HDPE lines exhibited lower tensile strength than their nylon counterparts, due to inherent material differences and residual stress from twisting, they demonstrated comparable elastic modulus values. Importantly, HDPE lines require significantly less energy during processing, offering a cost-effective and environmentally friendly alternative. Cutting tests showed that the 45° twisted HDPE line achieved cutting performance comparable to the square-profile nylon line and surpassed the round-profile variant. These results highlight the potential of HDPE as a viable, energy-efficient material for grass-cutting applications, particularly when optimized through geometric design.

## 1. Introduction

Backpack lawnmowers are becoming increasingly popular among homeowners and landscaping professionals because of their efficiency, portability, and suitability for maintaining outdoor areas. These mowers typically employ either rigid cutting blades or flexible polymer lines, each with different mechanical properties [1]. While metal blades are highly effective at removing dense vegetation and stubborn weeds, they pose a significant safety hazard when they come into contact with hard objects such as rocks, wood, or concrete [2,3]. Such impacts can result in fragmentation or ricochet, causing unpredictable debris trajectories that endanger both operators and bystanders. Similar findings have been reported in international contexts, where agricultural or landscaping equipment poses significant hazards due to high-speed blade impacts and projected debris [4,5].

In addition, blade fractures can occur upon impact, increasing injury risks [6]. According to data from Johns Hopkins University, approximately 6400 lawnmower injuries are treated in emergency rooms in the United States annually, with average treatment costs reaching $37,000 per case. Most of these injuries affect the waist and upper limbs (65.4%), followed by foot injuries (19.8%) [7,8,9,10]. A study in Lamphun Province, Thailand, reported 118 lawnmower injuries over a two-year period. A total of 87.5% of cases involved males with an average age of 36.5 years, and over half (56.2%) sustained eye injuries [11].

To minimize such risks, flexible polymer lines have emerged as a safer alternative to metal blades. Commercially available polymer lines are primarily made from engineering polymers such as nylon, which offer excellent mechanical strength and durability. Most commercially available grass-cutting lines come in square or round cross-sectional shapes and are typically produced from engineering polymers like nylon. These materials exhibit excellent mechanical properties but present significant challenges during processing. Nylon has a high forming temperature (typically above 240 °C) and contains amide groups (–CONH–) that result in a sharp melting transition, making it difficult to form complex profiles. Moreover, nylon is hygroscopic and must be thoroughly dried—typically at 80 °C for at least 4 h—before processing to prevent hydrolytic degradation [12,13]. In contrast, high-density polyethylene (HDPE), a commodity-grade polymer, offers better processability. It has a lower forming temperature starting from 180 °C and higher melt strength, particularly in extrusion grades used for blown film, which facilitates the formation of more complex shapes. HDPE is also non-hygroscopic, eliminating the need for pre-drying and thereby saving energy during processing [14,15], as shown in Table 1. However, nylon has significant processing disadvantages, including a high forming temperature over 240 °C and hygroscopic behavior, requiring pre-drying at 80 °C for several hours to avoid hydrolytic degradation during processing [16,17,18].

Its molecular structure, characterized by amide groups, contributes to sharp melting transitions and processing instability. These behaviors are well documented in polymer processing manuals. The hygroscopic nature and thermal sensitivity of nylon complicate extrusion and molding operations. In contrast, high-density polyethylene (HDPE), a general-purpose commodity polymer, offers several processing advantages. HDPE has a lower extrusion temperature (typically 180 °C), high melt strength suitable for blown film and profile extrusion, eliminating energy-intensive drying steps. While HDPE’s mechanical performance is generally inferior to that of nylon, it is significantly more energy-efficient and cost-effective to process [19,20,21].

In addition to material selection, the cutting performance in lawnmower systems is influenced by cutting angle and rotational speed. Prior research by O’Dogherty and Kakahy [8] emphasized the importance of blade geometry and speed, identifying optimal cutting angles between 25° and 40° to minimize energy consumption and maximizing efficiency. However, research into such geometric optimizations in polymer-based cutting lines is still limited. In particular, twisted or helical geometries—which can enable advantageous shear mechanics during rotation—still require thorough investigation with regard to cutting performance, durability, and wear behavior [22,23,24].

This study addresses this knowledge gap by developing and evaluating twisted grass-cutting lines fabricated from HDPE using a custom-designed extrusion die. The die enables precise formation of helical lines with twist angles of 0°, 15°, 30°, and 45°. The research investigates how twist angle affects the mechanical properties and cutting performance of the lines, while comparing the performance of twisted HDPE variants with commercially available nylon lines. Additionally, the study evaluates the energy consumption and wear resistance of each configuration to evaluate their suitability for industrial-scale production. By integrating material selection and geometric optimization, this work contributes to the design of safer, more sustainable, and cost-effective lawnmower components. The results help reduce the risk of operator injury, lower production energy requirements, and advance materials engineering for use in gardening equipment.

## 2. Materials and Methods

### 2.1. Materials and Preparation

**Figure 1:**
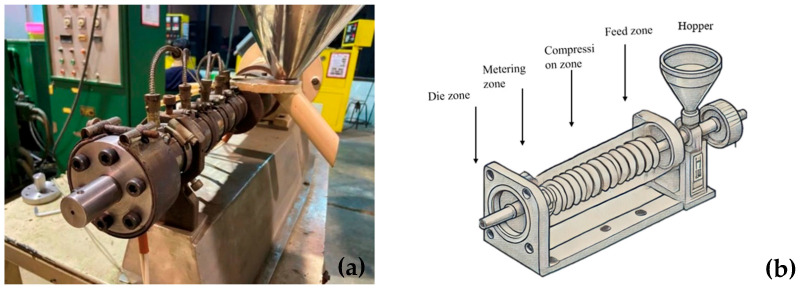
A photograph of the single-screw extrusion and twisting system used for producing twisted HDPE grass-cutting lines (**a**); a schematic illustration of the single-screw extruder showing the feed, compression, metering, and die zones (**b**).

**Figure 2:**
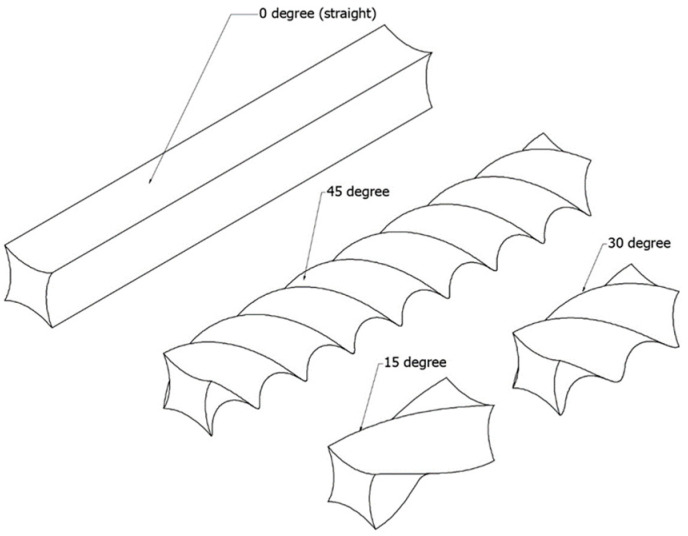
Grass-cutting line angles of 0°, 15°, 30°, and 45°.

High-density polyethylene (HDPE) resin, POLYMAXX grade H184J (Bangkok, Thailand), suitable for blown film applications, was processed using a single-screw extruder (Axon Process Supporting (PSE) Extruder, Breda, The Netherlands) with a screw diameter of 18 mm, as shown in Figure 1. A die specifically designed for extruding filaments was installed, equipped with a pulling and twisting mechanism to produce twisted filaments at angles of 0°, 15°, 30°, and 45°, as illustrated in Figure 2.

The temperature parameters of the single-screw extruder were adjusted for different zones, as shown in Table 2. Additionally, the tension speed and rotation speed were configured to achieve the specified twisting angles, as detailed in Table 3. Twisting of the HDPE lines was carried out immediately after extrusion, while the filaments remained in a semi-molten state. The temperature of the HDPE material during twisting was approximately 120–130 °C, measured at the die exit. Twisting was achieved by adjusting the relative speed between the rotating tension spool and the extrusion output. Once twisted, the lines were cooled and wound at ambient room temperature (25–27 °C) using a motor-controlled spooler system. These processing conditions ensured consistent twist angles and dimensional uniformity across all samples.

**Figure 3:**
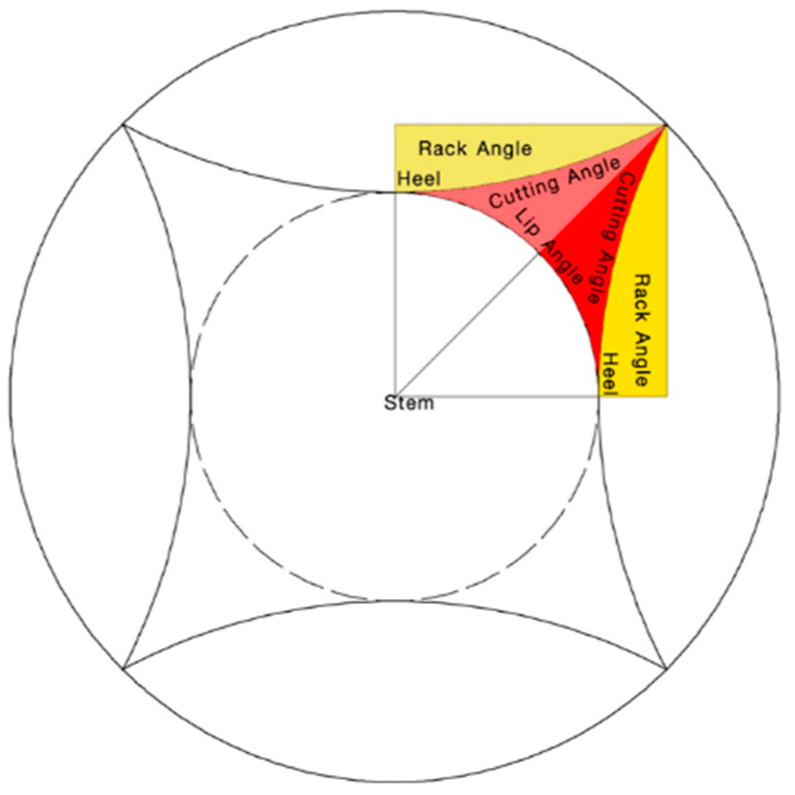
Design of cutting edges in grass-cutting lines.

**Figure 4:**
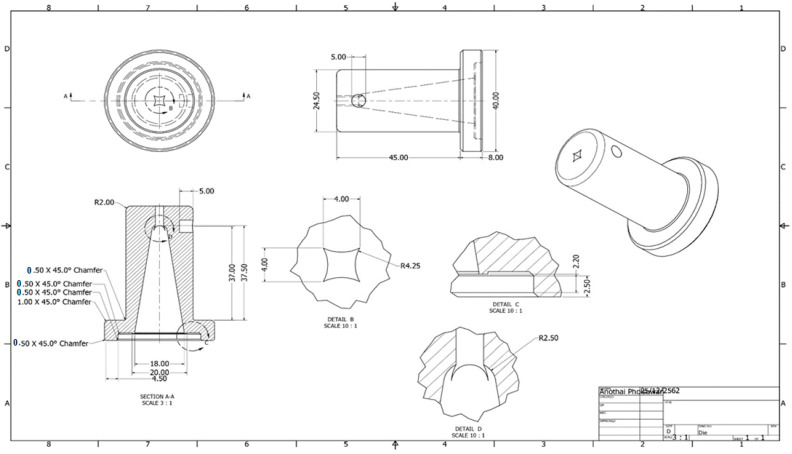
A die mold for forming grass-cutting lines.

The design of cutting dies is a crucial aspect of manufacturing precision components as it directly influences the quality and accuracy of the final product [25,26]. Figure 3 and Figure 4 illustrate the key design features of cutting dies, including the cutting angle, chip-breaking angle (rack angle), cutting edge support (lip angle), and cutting base (heel). These design parameters are carefully selected to optimize the cutting performance, minimize tool wear, and ensure consistent part geometry, as guided by the principles outlined in Tool Engineering and Design: Design of Single Point Cutting Tools.

Die molds for forming grass-cutting lines play a critical role in determining the final shape, strength, and performance characteristics of the lines. These molds are designed to control the extrusion process, ensuring the material is shaped correctly and uniformly while maintaining the desired properties such as tensile strength, flexibility, and durability. The design of the die mold involves careful consideration of several factors, including the material flow, die swell (the expansion of the material as it exits the die), and the desired final dimensions of the grass-cutting lines for HDPE (twisted lines, the die mold is specifically engineered to produce the required twist in the material while also managing the heat and pressure conditions that affect the properties of materials. The mold typically features a series of channels that direct the flow of the polymer material through the die, shaping it into the desired form. The geometry of the die, such as the angle of the cutting edge and the lip design, influences how the final product performs during cutting. Additionally, allowances for die swell are factored into the mold design to ensure the extruded lines meet the required specifications despite material expansion during extrusion.

In the case of twisted grass-cutting lines, the die is designed to impart a twist to the material during the extrusion process, which enhances the sharpness and cutting efficiency of the final product. The twist also contributes to the durability and strength of the line by providing additional surface area for cutting and reducing the risk of breakage during use. The overall goal of the die mold design is to optimize the manufacturing process, reduce waste, and produce grass-cutting lines that meet performance standards for safety, cutting efficiency, and longevity.

### 2.2. Characterization

The tensile strength of grass-cutting lines was evaluated by comparing commercially available grass-cutting lines with high-density polyethylene (HDPE) twisted lines. This comparison was carried out using a Universal Testing Machine (UTM) following ASTM D2256 standards, ensuring consistency and reliability in the results. The testing setup included capstan grips for securing the samples, with a load cell capacity of 10 kN. The distance between the grips was set to 250 mm, and the test was conducted at a pulling speed of 300 ± 50 mm/min. The testing temperature was controlled at 25 °C throughout the experiment to maintain consistency. Five specimens were tested. Using these standardized parameters, the study aimed to accurately assess the tensile properties of commercially available HDPE twisted grass-cutting lines. The results from this test will provide insights into the mechanical performance of the lines, specifically focusing on their tensile strength and durability, which are crucial factors in determining the effectiveness and safety of grass-cutting lines in real-world applications.

**Figure 5:**
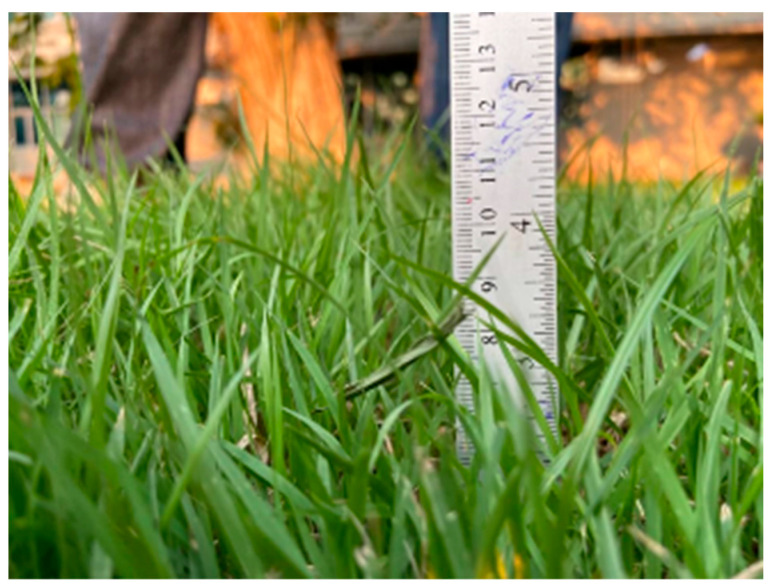
Initial grass height measured in the test plots before cutting trials. A standardized ruler was used to ensure uniform conditions across all samples.

The grass-cutting performance was evaluated using a backpack-type lawnmower, brand “Brush Cutter,” equipped with a 2-stroke engine capable of delivering a maximum power output of 2 horsepower at 10,000 rpm. A total of six testing plots were established, each containing grass with a height of 10 centimetres, as illustrated in Figure 5. Figure 5 shows the height of the grass in the testing plots. These plots were used to assess the effectiveness and efficiency of the grass-cutting lines under practical conditions. The objective was to evaluate how well the different types of grass-cutting lines performed in cutting grass, considering factors such as cutting speed, line durability, and overall performance in the field. This experimental setup was designed to simulate real-world lawnmowing conditions and provide valuable data for comparing the performance of HDPE twisted grass-cutting lines with commercially available products.

**Figure 6:**
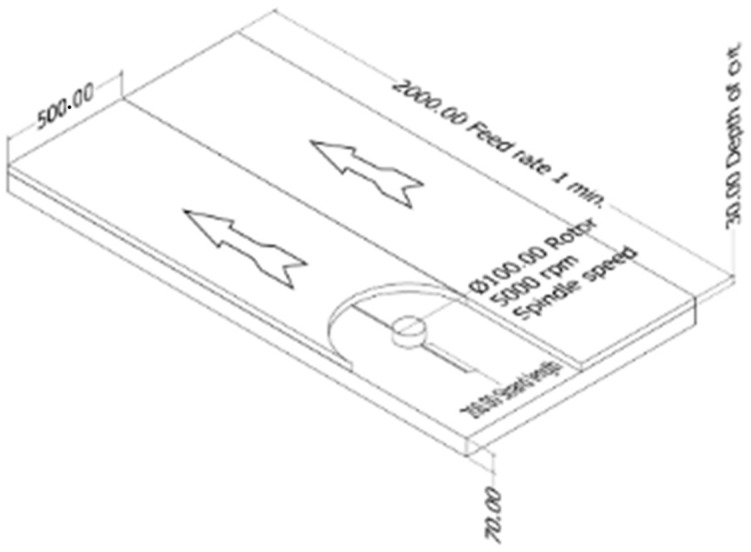
Characteristics of the grass test plot, parameters, and cutting direction. Note: “Ø” indicates diameter.

The cutting speed of the rotor spindle was set at 5000 rpm, with the grass-cutting lines installed at angles of 0° and 180° on the rotor. The testing plot for the grass cutting was 2 m × 1 m in size. The feed rate was set at 2000 mm/min, with a cutting depth of 30 mm. The cutting direction is shown in Figure 6.

After performing the cutting operation, the weight of the grass clippings was measured using a scale. To further analyze the performance of the grass-cutting lines, the lines and the clippings were examined under a light microscope (OM), Olympus model EM5 Mark II, equipped with a Zuiko macro lens with a focal length of 50 mm, Tokyo, Japan. This analysis aimed to assess the quality of the cut and the condition of the grass-cutting lines after use, providing insight into the wear and durability of the materials used. This method allowed for a comprehensive evaluation of the cutting efficiency, precision, and overall effectiveness of the twisted HDPE grass-cutting lines compared to conventional products.

The microstructures of the cutting lines were examined using a JEOL-6340F scanning electron microscope (SEM), operated under high-vacuum conditions at an accelerating voltage of 3.0 kV. The fractured surfaces were sputter-coated with a thin layer of gold to enhance surface conductivity. The wear performance was evaluated using a rotational wear test following ASTM D4060. Samples were tested at rotation speeds of 1000, 2000, and 3000 rpm using a standardized rotating disc method. This controlled setup enabled direct comparison of abrasion resistance by measuring weight loss, independent of field-based cutting variables.

## 3. Results

### 3.1. Macroscopic Structure of HDPE Twisted Grass-Cutting Lines

**Figure 7:**
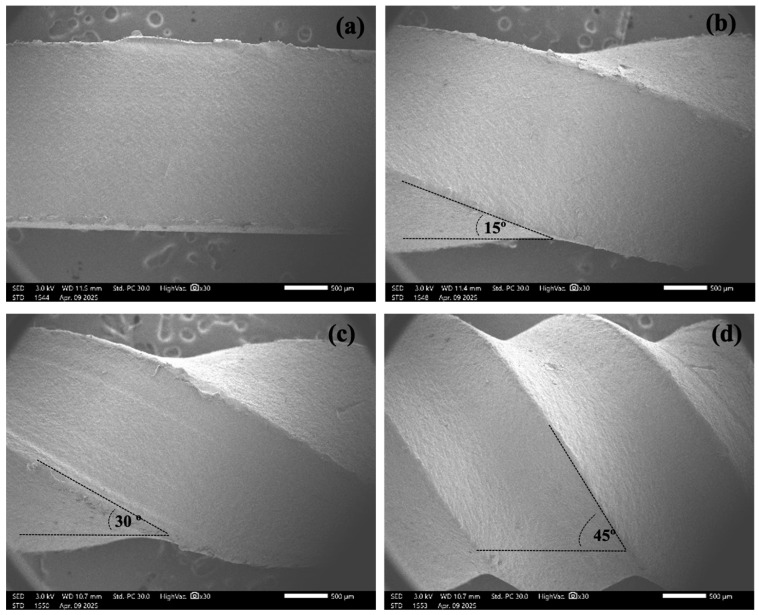
SEM images of grass-cutting line units with twisting angles of 0° (**a**), 15° (**b**), 30° (**c**), and 45° (**d**).; The twisting angle indicates the helical orientation of the cutting edge relative to the line axis.

The microstructure analysis of HDPE grass-cutting lines was conducted, and the twisting angles were calculated using the ImageJ software (1.54P version). The results are shown in Figure 7. The results indicated that the twisting angle deviated by ±2 degrees from the designated angle along the line length. This variation was attributed to the inconsistency in the extrusion speed of the line from the single-screw extruder. Additionally, during operation with the rotation device, wear was observed on the winding and twisting roller components due to pulling and twisting forces, further impacting the twisting angle.

### 3.2. Mechanical Property Comparison Between Commercially Available and Twisted HDPE Grass-Cutting Lines

The tensile properties of the grass-cutting lines are summarized in Table 4. Commercially available lines, including square and round profiles, demonstrated significantly higher tensile strength compared to the HDPE-based twisted lines. The square-profile nylon line exhibited the highest tensile strength at 350.39 ± 2.50 MPa, reflecting its excellent load-bearing capability. In contrast, the twisted HDPE lines showed tensile strengths of 36.60, 30.84, 35.70, and 39.24 MPa for twist angles of 0°, 15°, 30°, and 45°, respectively. Notably, the strength did not decrease linearly with increasing angle; instead, the 45° twisted line exhibited the highest tensile strength among the HDPE variants. The elastic modulus values of the HDPE lines remained relatively close across twist angles, with the maximum recorded at 45° (605.43 ± 2.31 MPa). While these were lower than the modulus of the nylon line (768.34 ± 1.80 MPa), the trend suggests that the twisting process introduces some structural stiffening. Regarding elongation at break, nylon again showed superior performance (346 ± 3%), reflecting its toughness and energy-absorbing capability. The twisted HDPE line at 45° recorded the highest elongation among the HDPE variants (40.50 ± 1.53%), and in general, elongation tended to increase with the twist angle. However, all HDPE values remained significantly lower than nylon, indicating intrinsic limitations in ductility.

The improvement in tensile strength and modulus in the 45° HDPE sample can be attributed to the alignment of molecular chains during twisting, which induces residual stress and enhances molecular orientation along the helical axis. This effect increases the resistance to deformation when stress is applied parallel to the twist, as more force is required to disentangle or separate aligned chains. This mechanism explains the observed mechanical enhancement at higher twist angles. These findings are consistent with Zhao et al. [27], who reported that mechanical performance in polymeric materials is sensitive to processing conditions and molecular orientation. When comparing the molecular structures, nylon contains polar amide groups (–CONH–), forming hydrogen bonds between chains that significantly increase mechanical strength. In contrast, HDPE consists of nonpolar saturated hydrocarbon chains (–CH_2_–CH_2_–), which are held together only by van der Waals forces. This fundamental difference explains the overall higher mechanical performance of nylon compared to HDPE [27,28,29].

Furthermore, attempts to twist the HDPE lines beyond 45° led to premature failure and breakage during fabrication. This is attributed to the structural limitations caused by excessive twisting, which distorts the alignment of polymer chains. Under standard conditions, molecular chains are oriented along the axial direction to maximize tensile strength. As twist angles increase, the chains are forced into oblique orientations, relying more on weaker transverse secondary bonds rather than covalent bonds along the polymer backbone. This shift results in reduced tensile strength and structural instability. These observations align with the findings of Rao and Farris [30], who demonstrated that while moderate twisting improves fiber strength, excessive twisting induces damage and reduces mechanical performance.

This study evaluated the cutting performance of various grass-cutting line designs by measuring the mass of grass removed within a standardized cutting time of two minutes per plot. The results, shown in Table 5, compare the performance of commercially available nylon lines with twisted HDPE lines at different twist angles.

Among the tested samples, the twisted HDPE line with a 45° angle achieved the highest grass-cutting efficiency, removing 210 g of grass, surpassing the square-profile nylon line (203 g). The 15° and 30° twisted lines also performed well, while the 0° HDPE line cut the least amount of grass. These results indicate that increasing the twist angle enhances cutting performance, likely due to the introduction of shear mechanics at the cutting interface. In comparison, the square-profile nylon line outperformed traditional round-profile lines in previous studies by Karker and Velinsky [31], which attributed improved performance to the larger cutting surface and sharper edge geometry. Although the round-type line was not tested here, the square nylon line serves as a strong benchmark.

**Figure 8:**
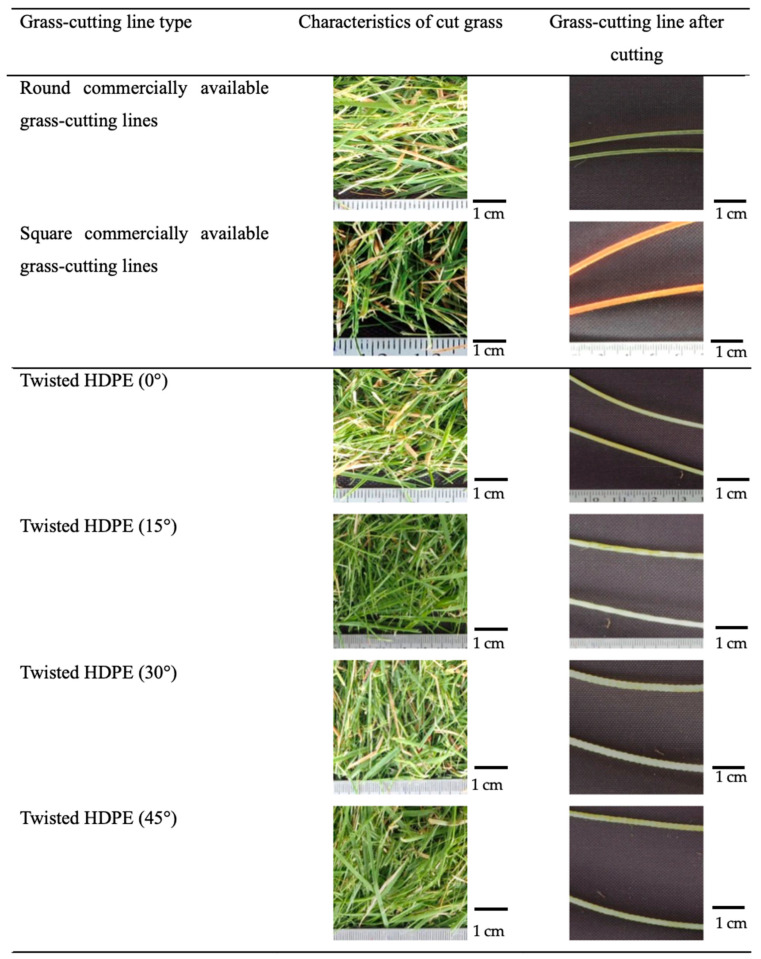
Grass-cutting lines before (**left**) and after cutting (**right**).

Observations from the cut grass morphology in Figure 8 revealed that twisted HDPE lines caused complete fragmentation of the grass blades, particularly at higher twist angles. It should be noted that the orange/yellow color observed on the square-profile commercial nylon line is not a result of use but reflects the original product color intended by the manufacturer to enhance visibility and brand identification. This is attributed to the shear stress generated by the twisted geometry during rotation. The polyethylene material’s lower friction coefficient than nylon’s also contributed to a cleaner cut, driven more by shear than friction. The cutting edge formed by the twisted structure acts more like a slicing mechanism, increasing penetration and fragmentation. These findings suggest that the 45° twisted HDPE line offers a viable and even superior alternative to commercial nylon lines in terms of cutting performance. The results also demonstrate that geometric optimization and appropriate material selection can significantly improve functional performance in real-world applications.

In addition to its mechanical properties, HDPE demonstrates a notably lower carbon footprint than polyamide (PA), primarily due to its reduced energy consumption during manufacturing. HDPE production generates 55–58% less carbon dioxide emissions than PA, highlighting its environmental superiority. Economically, HDPE is markedly more cost-efficient, with production costs approximately 72.18% lower than those of PA [32,33,34]. This cost difference underscores its viability for large-scale applications in grass-cutting line manufacturing. From both environmental and economic standpoints, the replacement of PA with HDPE in the production of grass-cutting lines offers considerable advantages. Such substitution not only decreases CO_2_ emissions but also significantly reduces manufacturing expenses, promoting sustainable industrial practices. Furthermore, the high recyclability of HDPE presents an added benefit, contributing to more effective plastic waste management and aligning with global efforts toward environmental sustainability.

### 3.3. Wear Resistance of Grass-Cutting Lines

**Figure 9:**
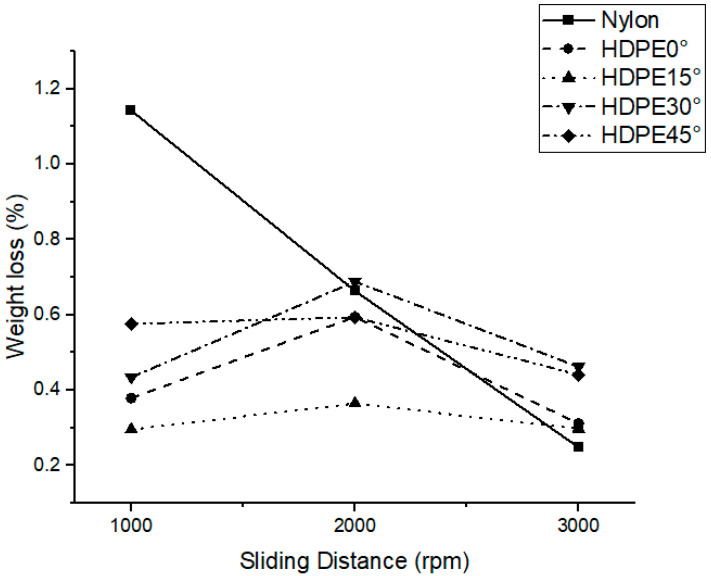
Weight loss comparison between the commercial nylon cutting line and twisted HDPE grass-cutting lines at different twist angles.

The wear resistance of the grass-cutting lines was evaluated by measuring the weight loss after testing, comparing commercially available nylon lines with twisted HDPE lines at different twist angles. The results are illustrated in Figure 9. It was observed that the nylon lines exhibited the highest weight loss across all testing intervals. However, as the test duration increased, the rate of wear appeared to decrease. This behavior may be attributed to the formation of a lubricating transfer film on the contact surface or to thermal-induced surface hardening. As described in tribological studies by Bahadur [35] and Jain and Bahadur [36], polymers undergoing sliding contact often transfer fragments to the counterface, forming a thin film that acts as a self-lubricating layer. This phenomenon is more prominent in polymers with low cohesive energy density (CED). Nylon, having higher CED and stronger intermolecular bonding compared to HDPE, tends to form more stable transfer films during frictional contact. This can help reduce wear over time, despite initial losses, and may explain the observed reduction in wear rate at extended test durations.

Specifically, localized heating during frictional contact may have caused partial cross-linking in the surface layer of nylon. This could involve chain scission followed by recombination of amide groups, resulting in a tougher surface and improved wear resistance in the later stages of the test.

**Figure 10:**
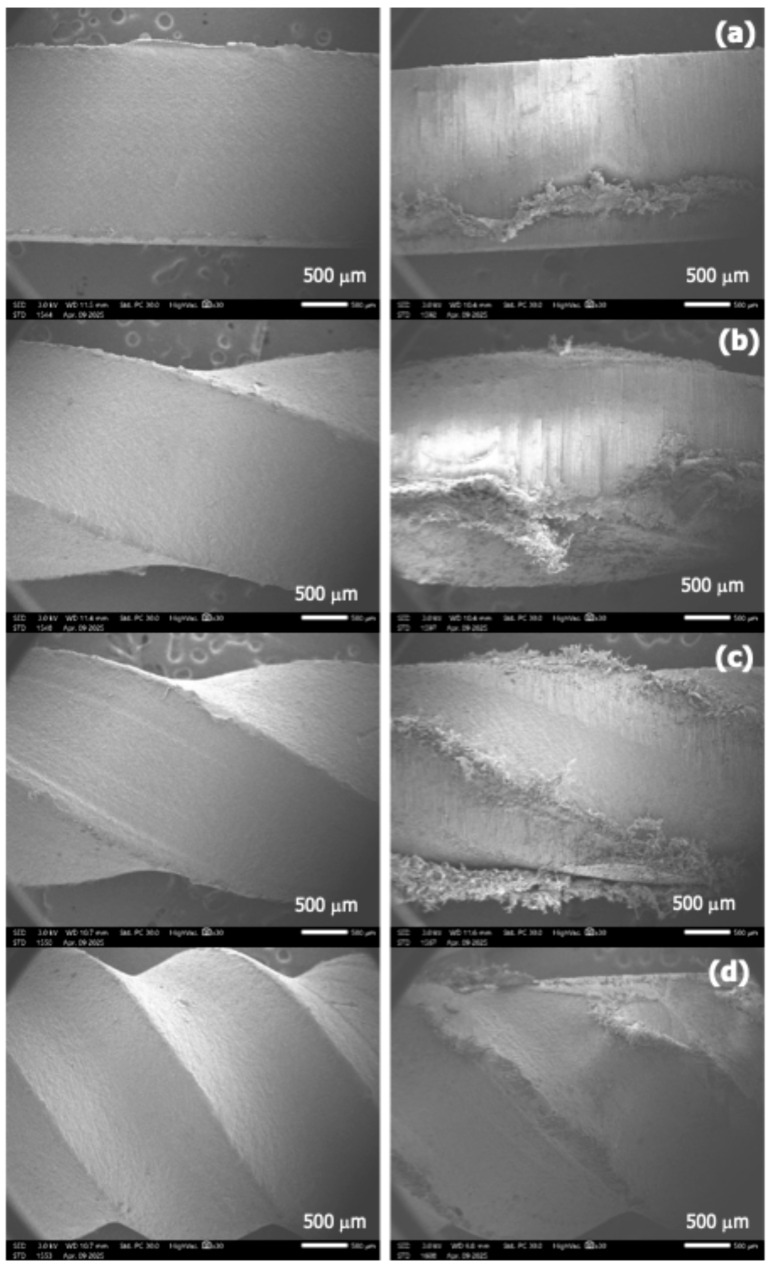
SEM images of surface morphology before (**left**) and after (**right**) wear testing at 3000 rpm for twisted HDPE cutting lines with different twisting angles, 0° (**a**), 15° (**b**), 30° (**c**), and 45° (**d**). Each pair of images shows the same line unit to compare surface changes due to wear.

In contrast, the twisted HDPE lines showed significantly lower weight loss compared to the nylon line at every test interval. Among the HDPE variants, the 15° twisted line demonstrated the least weight loss throughout the entire test range. This suggests that the reduced contact area and altered surface geometry from twisting effectively decreased friction and, thus, wear. A general trend was noted for the HDPE samples: weight loss peaked at 2000 rpm and decreased at 3000 rpm. This behavior may be linked to thermal effects or changes in the dominant wear mechanism. At lower speeds, abrasive wear appears to dominate, while at higher speeds, a transition to adhesive wear may occur. The corresponding surface morphologies before and after testing, shown in Figure 10, support this interpretation

## 4. Conclusions

This study successfully developed and evaluated a custom-designed mold for producing twisted grass-cutting lines from high-density polyethylene (HDPE) with twist angles of 0°, 15°, 30°, and 45°. The mold demonstrated excellent geometric precision, enabling consistent formation of twisted profiles and confirming the feasibility of shaping HDPE into complex filament structures. The ability to control twist geometry was shown to be critical, as it significantly influenced the mechanical properties and cutting performance of the resulting lines. Although commercial nylon lines exhibited superior tensile strength and elongation due to their hydrogen-bonded amide molecular structure, the 45° twisted HDPE lines achieved a remarkable balance between mechanical integrity and cutting efficiency. Notably, the 45° HDPE line delivered cutting performance comparable to square-profile nylon and outperformed round-profile nylon lines, demonstrating that geometric optimization can partially offset HDPE’s material limitations. Additionally, wear resistance tests revealed that twisted HDPE lines, particularly at 15°, experienced lower material loss than nylon during rotational abrasion, suggesting improved durability under certain operational conditions. These results, coupled with HDPE’s significantly lower energy requirements and cost-effective processing, underscore its potential as an energy-efficient and sustainable alternative to nylon for grass-cutting applications. However, it is important to acknowledge that this study focused on initial performance metrics under controlled conditions. Long-term durability, including cyclic loading, environmental exposure, and fatigue resistance, was not within the current scope and remains an important avenue for future research. Continued investigation into reinforcing strategies, such as filler incorporation or surface modification, may further enhance HDPE’s mechanical properties while retaining its processing and environmental advantages. Such developments could expand the applicability of twisted HDPE lines across a broader range of consumer and industrial lawn maintenance tools.

## Figures and Tables

**Table 1:** A comparison of HDPE and Nylon 6 properties [14,15].

Properties		
Functional Groups	(–CH_2_–CH_2_–)	(–CONH–)
Mechanical Properties		
Tensile Strength (MPa)	20–37	60–100
Hardness (Shore D)	55–70	75–85
Coefficient of Friction (COF)		
- Static COF	0.20–0.30	0.25–0.35
- Dynamic (Kinetic) COF	0.10–0.22	0.25–0.40
Thermal Properties		
Melting Point (°C)	180	240
Specific Heat (J/kg·K)	2300	1700
Latent Heat of Melting (J/kg)	240	160
Price (USD per kg)	1.11	2.04

**Table 2:** Parameters for single-screw extruder setup.

Zone	Temperature (°C)
Feed Zone	140
Compression Zone	150
Metering Zone	160
Die Zone	180

**Table 3:** Parameters for twisting angle formation.

Angle (°)	Tension Speed (rpm)	Rotation Speed (rpm)
0	35	0
15	35	10
30	35	20
45	35	35

**Table 4:** Mechanical property comparison between commercially available and twisted HDPE grass-cutting lines.

Type of Grass-Cutting Line	Tensile Strength (MPa)	Elongation (%)	Elastic Modulus (MPa)
Square (Commercially Available)	350.39 ± 2.50	346 ± 3	768.34 ± 1.80
Twisted HDPE (0°)	36.60 ± 1.40	619.47 ± 0.84	36.50 ± 2.26
Twisted HDPE (15°)	30.84 ± 1.30	476.9 ± 1.09	19.31 ± 1.54
Twisted HDPE (30°)	35.70 ± 0.93	604.36 ± 4.71	34.63 ± 1.04
Twisted HDPE (45°)	39.24 ± 1.27	605.43 ± 2.31	40.50 ± 1.53

**Table 5:** Weight of grass cut from test plots comparison between commercially available and twisted HDPE grass-cutting lines.

Grass-Cutting Line Type	Weight of Cut Grass (g)
Commercially available grass-cutting lines	
Square	203
Twisted grass-cutting lines	
Twisted HDPE (0°)	105
Twisted HDPE (15°)	164
Twisted HDPE (30°)	133
Twisted HDPE (45°)	210

## Data Availability

Data are contained within the article.
